# Micelle-template synthesis of hollow silica spheres for improving water vapor permeability of waterborne polyurethane membrane

**DOI:** 10.1038/srep46638

**Published:** 2017-04-21

**Authors:** Yan Bao, Tong Wang, Qiaoling Kang, Chunhua Shi, Jianzhong Ma

**Affiliations:** 1 College of Bioresources Chemical and Materials Engineering, Shaanxi University of Science and Technology, Xi’an 710021, China; 2 Shaanxi Research Institute of Agricultural Products Processing Technology, Xi’an 710021, China; 3 Key Laboratory of Leather Cleaner Production, China National Light Industry, Xi’an 710021, China

## Abstract

Hollow silica spheres (HSS) with special interior spaces, high specific surface area and excellent adsorption and permeability performance were synthesized via micelle-template method using cetyl trimethyl ammonium bromide (CTAB) micelles as soft template and tetraethoxysilane (TEOS) as silica precursor. SEM, TEM, FT-IR, XRD, DLS and BET-BJH were carried out to characterize the morphology and structure of as-obtained samples. The results demonstrated that the samples were amorphous with a hollow structure and huge specific surface area. The growth of HSS was an inward-growth mechanism along template. Notably, we have provided a new and interesting fundamental principle for HSS materials by precisely controlling the ethanol-to-water volume ratio. In addition, the as-obtained HSS were mixed with waterborne polyurethane (WPU) to prepare WPU/HSS composite membrane. Various characterizations (SEM, TEM, FT-IR and TGA) revealed the morphology, polydispersity and adherence between HSS and WPU. Performance tests showed that the introduction of HSS can improve the water vapor permeability of composite membrane, promoting its water resistance and mechanical performance at the same time.

Polyurethane (PU) is widely used in coating industry for its excellent wear resistance, low temperature flexibility, and good physical and chemical stability^1^,^2^,^3^. However, the PU membrane suffers several defects, such as yellowing, poor permeability, and the release of organic solvent, which hinder its further development. Specifically, the poor permeability will lead to low air permeability and water vapor permeability, so as to severely degrade sanitation performance^4^. Primarily, the water vapor permeability is of great importance when PU membrane is used as the coating of synthetic leather for shoes and garments^5^. It allows the sweat to evaporate promptly, especially when human bodies are in hot environments. Moreover, in order to adapt to the development of coating industries, waterborne polyurethane (WPU) with outstanding comprehensive performance and environmentally-friendly characteristics is generally taking place of traditional PU. Even though the water vapor permeability of WPU shows a slight promotion, but still in poor condition and the mechanical property dropped simultaneously.

Previous researches have shown that there are several important factors affecting the water vapor permeability of polymer membrane^6^. Among them, hydrophilic nature of polymer chain^7^ and free volume in polymer membrane^8^ are the decisive forces. First and foremost, from a chemical point of view, hydrogen bonds will be formed between water vapor molecules and hydrophilic groups in polymer membrane. With the vibrating of polymer chains, water vapor molecules diffuse from a region of greater to one of less concentration^9^. Furthermore, from a physical point of view, the free volume theory is widely applied to expound the relationship between mass transfer in a polymer film and water vapor dependence on number, shape and size of free volume^10^.

The above statements have been verified by theoretical analysis and experimental results. In Mondal *et al*.’s work^11^, they observed that water vapor permeability of PU was influenced by presence of both hydrophilic groups content as well as soft segment crystal melting. Zhang *et al*.^12^ reported a method of adding white wood flours with different ratio to polyurethane, and the experimentation results showed that the air and water vapor permeability of polyurethane membrane were obviously enhanced.

However, the generalisability of much published research on this issue is problematic. Most studies on the filler materials of PU membrane have only focused on solid materials of macro-range and overlooked hollow micro-nano materials with mesoporous structure^13^, which resulted in an insufficient performance improvement. The usage of inorganic nanoparticles would be desirable filler materials to overcome the aforementioned limitations. Because, polymer/inorganic nanocomposites showed a series of preeminent functionalities as compared with conventional polymer composites, such as improved mechanical strength, wear resistance, yellowing resistance, water resistance, antimicrobial behavior, self cleaning, hygienic properties and so on.

Among a variety of inorganic nanoparticles, hollow structured silica spheres have been widely concerned in the past few years due to the ingenious combination of hollow architecture with the excellent characteristics of silica materials^14^,^15^,^16^. Benefitting from the merits of low density, large specific surface area, the huge void space, special photoelectric properties and good biocompatibility^17^, hollow silica spheres (HSS) present promising application prospects in catalysis^18^, waste removal^19^, adsorption, storage^20^, and sustained/controlled release of agents with various functionalities^21^,^22^,^23^,^24^,^25^,^26^. Different methods have been employed to prepare HSS, including template-assisted synthesis^27^,^28^, self-templating method^29^,^30^, spray drying^31^,^32^ and hydrothermal synthetic method^33^,^34^. Among them, templating method is one of the most commonly used approaches. Usually, core-shell structured spherical composites are synthesized by coating silica on spherical template cores such as polymer based templates^35^,^36^,^37^, inorganic templates^38^,^39^,^40^ and emulsion templates^41^. Then, the template cores are removed by either high-temperature calcination in air or chemical etching with solvent to obtain hollow structure. However, this is time-consuming and may cause air pollution and the collapse of hollow structure. Therefore, there is a great challenge to develop a simple, effective and environmental friendly way for synthesizing HSS^42^.

Micelle template method is used as an alternative approach wherein micelle templates are formed and silica is deposited on templates^43^,^44^. In this article, HSS with regular structure, hollow cavity, narrow particle size distribution and amorphous shell were conveniently fabricated by micelle template. The innovation points of our research were that we employed cetyl trimethyl ammonium bromide (CTAB) micelle as cavity and mesoporous templates, which was very easy to prepare and remove at ambient surrounding. In contrary to current soft template synthesizing strategies including emulsion or micro-emulsion templates, micelle template method had improved structural stability and monodispersity. Significantly, we systematically demonstrated the influence of factors on morphology by changing the synthetic parameters, such as CTAB dissolving methods and ethanol-to-water volume ratio. And the possible mechanism of morphological change of HSS was proposed according to the research results. Finally, the as-prepared HSS were introduced into WPU emulsion by physical blending method to prepare WPU/HSS composite emulsion and composite membrane. Importantly, the multi-functional composite membrane have potential applications in improving water vapor permeability of waterborne polyurethane coating.

## Experimental

### Materials

Tetraethoxysilane (TEOS) and cetyl trimethyl ammonium bromide (CTAB) were purchased from Tianjin Kemiou Chemical Reagent Co., LTD. Ammonia (25 wt% NH_3_ in water) was supplied by Tianjin Fuyu Fine Chemical Co., Ltd. Ethanol was obtained from Tianjin Hedong Hongyan Chemical Reagent Factory. All the reagents were of analytical grade and used as received without further purification. WPU (technical pure) was provided by Yantai Daocheng Chemical Co., Ltd. The deionized water was from our laboratory.

### Preparation of hollow silica spheres

HSS were prepared via the hydrolysis and condensation of TEOS in an aqueous basic-ethanol medium. Typically, 0.08 g of CTAB was dissolved in 50 mL of mixed aqueous solution of water and ethanol with stirring. Then 0.5 mL of TEOS and 0.5 mL of ammonia were added sequentially. The resulting mixture was further stirred at 30 °C for 6 h to give a white suspension. After reaction, the suspension were centrifuged and washed with water and ethanol by two cycles to remove any residual organics and ammonia. The final particles were collected after drying over-night at 60 °C.

### Preparation of WPU/HSS composite emulsion and composite membrane

WPU/HSS composite emulsion was obtained by physical blends of WPU resin and HSS. First, 0.09 g of HSS was dispersed in 15 mL of aqueous solution containing one-third of ethanol with 20 min ultrasonation. Then the dispersion was added into a 100 mL three-necked flask containing 30 g of WPU resin. The blending process was kept at 75 °C for 6 h with stirring.

20 g of WPU/HSS composite emulsion was poured into a square glass tank and laid on the horizontal surface until dried completely at ambient temperature to obtain WPU/HSS composite membrane.

### Morphological and structural characterization for HSS and WPU/HSS composite membrane

The morphology of HSS and WPU/HSS composite were observed by Scanning Electron Microscope (SEM, S48000, Japan) and Transmission Electron Microscope (TEM, Hitachi, H-7650, Japan). For SEM, the HSS were scattered on the conductive adhesive of sample platform and sputter-coated with gold. The WPU/HSS composite membrane were breaked off in liquid nitrogen and then attached to the samples platform and sputter-coated with gold. For TEM, the samples were prepared by diluting HSS or composite emulsion with ethanol and dropping the obtained suspension on a copper grid.

The chemical structure of HSS and WPU/HSS composite membrane were analyzed by Fourier Transform Infrared Spectroscopy (FTIR, VECTOR22, Brucker, Germany). The powder samples were pressed into a transparent sheet together with potassium bromide (KBr) by a mould, whereby the samples and KBr should be dry.

Wide-angle X-ray Diffraction (XRD, Rigaku, D/max-2200, Japan) was used to determine the crystalline phase of HSS. Radial scans of intensity versus scattering angle were recorded from 10° to 70° using a Cu Kα radiation. And the samples were flattened in a sample container by a glass slide.

The average particle size distribution of micelles and HSS were obtained by dynamic light scattering (DLS) measurements at 25 °C using a Zetasizer NanoZS (Malvern Instruments Ltd., UK).

The specific surface areas of HSS were measured with Brunauer-Emmett-Teller (BET) and Adsorption Analysis. Pore size distributions were calculated from desorption branches of isotherms by the Barrett-Joyner-Halenda (BJH) method. Before test, the powder samples were degassed at 300 °C under vacuum for 3 h.

Thermogravimetric analysis (TGA) curves of WPU/HSS composite membrane were carried out in a Perkin-Elmer thermogravimetric analyzer under air stream. The temperature gradient started from 25 °C to 600 °C at a heating rate of 10 °C/min.

### Performance tests of WPU/HSS composite membrane

#### Water vapor permeability of WPU/HSS composite membrane

Water vapor permeability (WVP) of composite membrane was measured by permeability cup method, a National Standard test method in China. First, a permeability cup with area of 10 cm^2^ was filled with 30 g of deionized water. Then the composite membrane was sealed on the top of the cup. Keep the cup in an incubator with constant temperature (37 °C) and humidity (90% RH) for 24 h. The water vapor permeability was valued by the following formula:


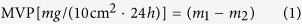


where 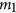 and 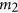 are the total mass of permeability cup, composite membrane and water, weighted up promptly and after 24 h respectively.

#### Water uptake of WPU/HSS composite membrane

Three squares with size of 1.5 cm × 1.5 cm were clipped from composite membrane and immerged in distilled water for 24 h. Each one was weighed on analytical scale before and after immersion, denoted as *m*_*0*_ and *m* respectively. Water absorption (*W*) was calculated according to the following formula and 3 data were averaged for a more convincing result:


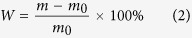


#### Mechanical property of WPU/HSS composite membrane

Tensile strength and elongation at break of composite membrane was measured using a functional materials examination machine made by Taiwan High Iron Science and Technology Stock Company with extending speed of 500 mm/min and backhaul speed of 100 mm/min. Composite membrane was sampled with the standard dumbbell-like mould. And before examination, all the samples were placed in desiccator of concentrated sulfuric acid for 24 h for air conditioning.

## Results and Discussion

### Morphological and structural characterization of HSS

**Figure 1:**
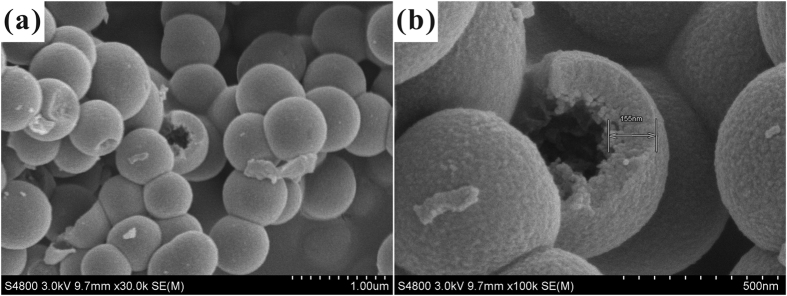
SEM images of HSS: (**a**) Low magnification, (**b**) High magnification.

Low-magnification SEM image (Fig. 1a) showed that the HSS samples were uniform in size with spherical shape, and the average size was ~500 nm. Broken spheres prove the hollow nature of silica samples. Closer examination (Fig. 1b) indicated that the silica shell was rough, composed of many tiny silica particles. The thickness of the shell was about 150 nm. These constituent silica particles were about 20 nm in dimension, attaching to each other to form a relatively dense silica shell. The HSS could not be destroyed into individual silica particles even after fully grinded. So, strong chemical bonding is believed to be formed between the contacting lateral surfaces. Small-sized constituent silica particles are inclined to attach to each other to minimize the surface energy, forming hollow silica shell. Also for this reason, the adjacent HSS may further coagulate to form the twin-like structure, as observed in Fig. 1(a).

**Figure 2:**
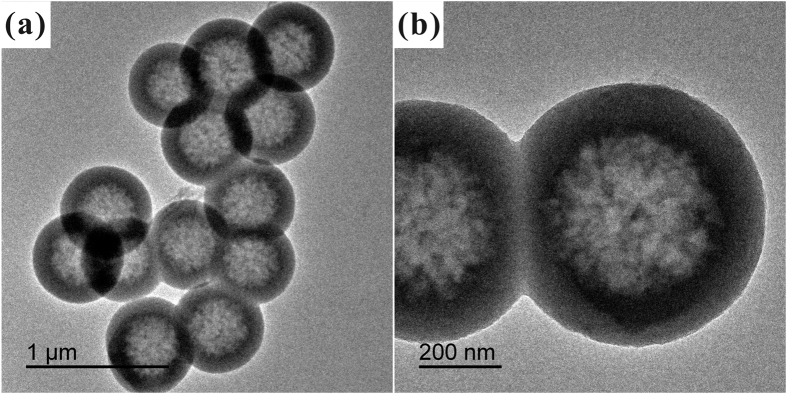
TEM images of HSS: (**a**) Low magnification, (**b**) High magnification.

To furher confirm that HSS possess apparent hollow structure, the HSS samples were observed by TEM. As shown in Fig. 2, the typical TEM images of HSS clearly revealed that all those samples fabricated by micelle-template method were hollow in character and the shell thickness of the spheres was about 150 nm. Meanwhile, the outer surface of HSS was smooth, while the inner surface was rough. These tendencies are same with the results of SEM observations.

**Figure 3:**
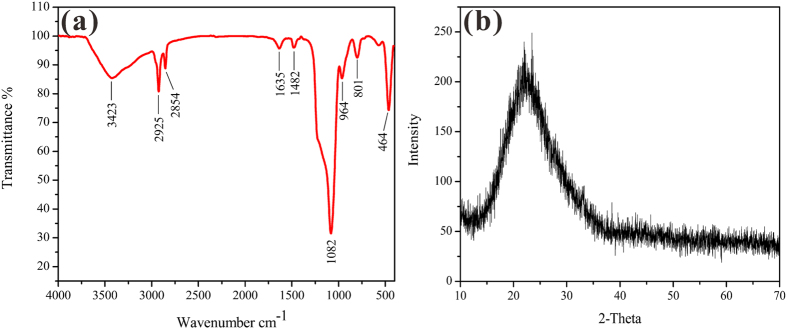
(**a**) FT-IR spectrum of HSS, (**b**) XRD pattern of HSS.

Figure 3(a) showed the FT-IR spectrum of the obtained HSS samples. A distinctive Si-OH stretching vibration was displayed at about 3423 cm^−1^. The strong absorption peak at 1082 cm^−1^ was belonged to Si-O-Si asymmetric stretching. The peaks at 801 cm^−1^ and 464 cm^−1^ were assigned to symmetric stretching vibration of Si-O, and the peak at 964 cm^−1^ was due to bending vibration absorption of Si-OH. Peak around 1635 cm^−1^ was the bending vibration peak of H-O-H in water. The peaks at 2925 cm^−1^ and 2854 cm^−1^ were attributed to asymmetrical stretching vibration of -CH_3_ and -CH_2_-, which may be caused by the residual organic matter inside the samples. These results demonstrated that the as-obtained sample is silica. In addition, the powder XRD pattern in Fig. 3(b) showed a broad diffraction peak between 12° and 38°, indicating that the silica samples are of amorphous structure. This amorphous structure endowed HSS with loose and permeable silica shells.

### The influence of CTAB dissolving methods on the morphology of HSS

**Figure 4:**
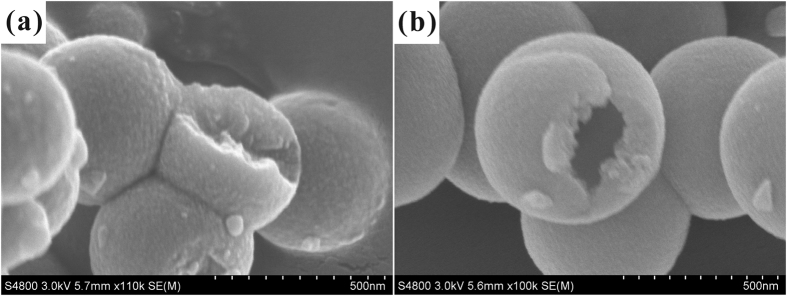
SEM images of hollow silica spheres as a function of CTAB dissolving methods: (**a**) CTAB was dissolved in water, (**b**) CTAB was dissolved in mixed aqueous solution of water and ethanol.

To elucidate the importance of ethanol on the whole reaction system, the control experiment was carried out that using pure water as the initial solvent of CTAB. The SEM images (Fig. 4a) showed that when CTAB was dissolved in pure water, only nearly solid silica spheres were obtained with a smaller particle size. Because, when CTAB was dissolved in pure water system, the hydrophobic carbon chain with lower dielectric constant escaped from the water medium rapidly and entangled with each other. Then the smaller diameter micelles were formed and the surface energy of the system would be dropped to a minimum. On the other hand, the addition of ethanol was in favor of the formation of hollow structure. This is due to the fact that ethanol has lower dielectric constant than water. While ethanol and water were used as co-solvents, it is easier for ethanol to seep into the interior of the CTAB micelle to enlarge it, forming a rich-ethanol phase inside micelle and a rich-water phase outside micelles. Then the expanded micelle act as templates to direct the hydrolysis and assembly of silica precursor. The internal hydrophobic region of CTAB micelle allowed the organic precursor in and then provided place for precursor to hydrolyze. When exposed to alkaline condition, precursor hydrolyzed rapidly to form tiny silica seeds. Since negatively charged, these silica seeds attracted to the inner surface of positively charged CTAB micelle by electrostatic interactions, thus resulting a silica shell. After complete hydrolysis of precursor, HSS could be obtained (Fig. 4b). So, larger micelle is more conducive to the formation of hollow structure.

### The influence of ethanol-to-water volume ratio on the morphology of HSS

**Figure 5:**
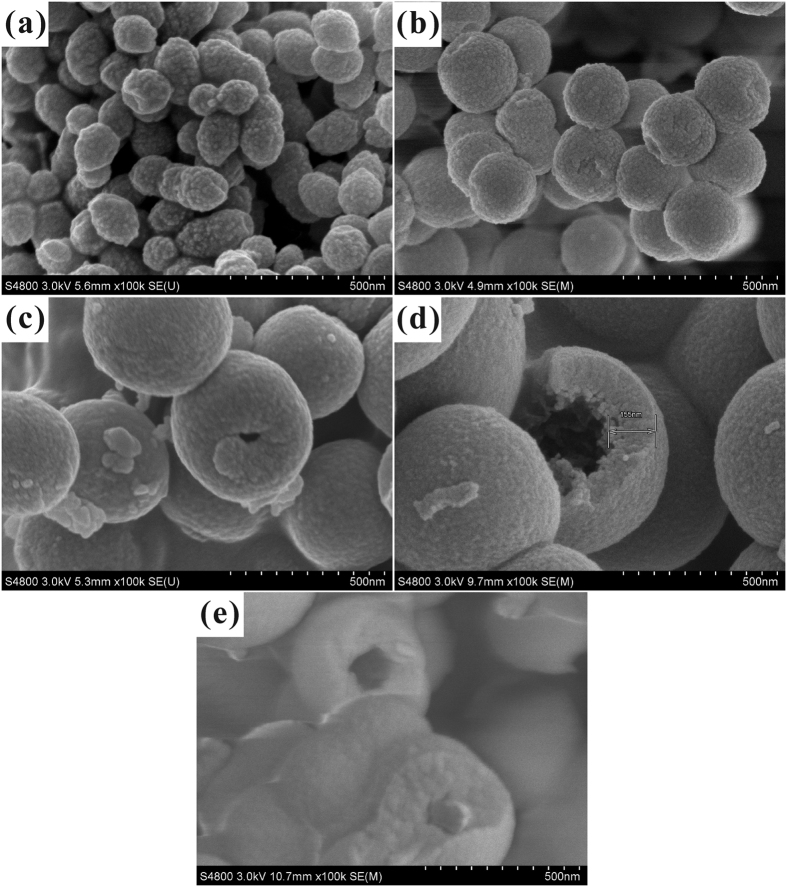
SEM images of hollow silica spheres at different ethanol-to-water volume ratio: (**a**) 0.3, (**b**) 0.4, (**c**) 0.5, (**d**) 0.6, (**e**) 0.7.

**Figure 6:**
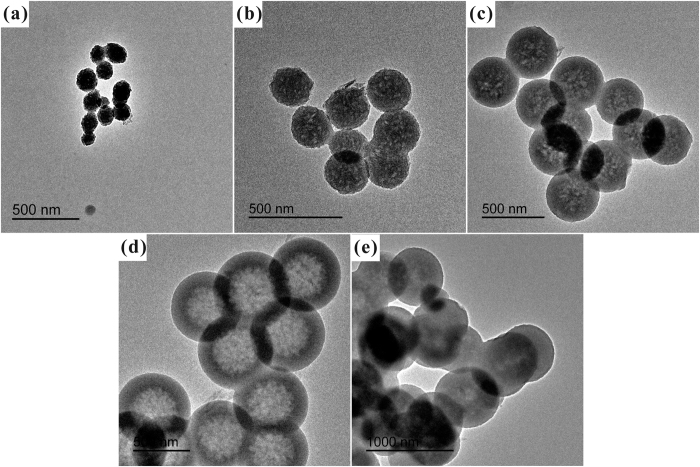
TEM images of hollow silica spheres at different ethanol-to-water volume ratio: (**a**) 0.3, (**b**) 0.4, (**c**) 0.5, (**d**) 0.6, (**e**) 0.7.

Figs 5 and 6 showed the SEM and TEM images of a series of HSS prepared at different ethanol-to-water volume ratio (R_e/w_), respectively. Diameters of HSS increased gradually from 150 nm to 600 nm when the R_e/w_ changed from 0.3 to 0.6. Furthermore, the broken or fractured HSS in SEM images together with TEM images permit us to observe the inner space directly, indicating that the solid silica spheres were transformed to hollow silica spheres by increasing R_e/w_ from 0.3 to 0.6. In this period, the hollow silica surface became smoother. The above morphological evolutions may due to the following reasons. When more ethanol was added, dielectric constant of the system reduced. More ethanol would enter into CTAB micelles, enlarging the template, to form larger HSS. Meanwhile, more TEOS species would enter into CTAB micelles together with ethanol due to increased solubility of TEOS in the rich ethanol system. The presence of ethanol also reduced the hydrolysis rate of precursor, creating HSS with smoother surface. However, further increasing the R_e/w_ to 0.7, the ethanol content in the rich-water phase rose and the miscibility between TEOS and water increased greatly. A portion of TEOS was dissolved in rich-water phase outside the micelle, rather than into the micelle interior to further increase the template size. So, HSS with smaller hollow interior cavity and nearly invariable particle size were obtained, as shown in Figs 5(e) and 6(e).

**Figure 7:**
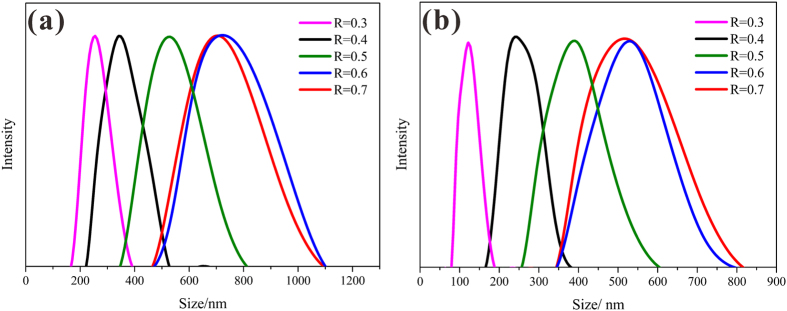
DLS plots of (**a**) hollow silica spheres and (**b**) micelle at different ethanol-to-water volume ratio. (R_e/w_ = 0.3, 0.4, 0.5, 0.6 and 0.7, respectively).

It is known that the size of particles synthesized in a mixed aqueous solution of water and ethanol are governed by the amount of ethanol. Figure 7(a) revealed the hydrodynamic particle size distribution of HSS obtained at different R_e/w_, suggesting the increasing hydrodynamic diameter of HSS from 250 nm to 800 nm with the R_e/w_ increasing from 0.3 to 0.6. Interestingly, it reached to a stable state with R_e/w_ keeping on going up to 0.7. As a control, Fig. 7(b) showed the systematic DLS of micelle (CTAB) by varying R_e/w_, which indicated the similar tendency with Fig. 7(a). However, in contrast to the actual size in SEM and TEM images, all the samples in DLS results showed a larger population of particles with a large diameter, due to the little conglomeration and instability of particles.

**Figure 8:**
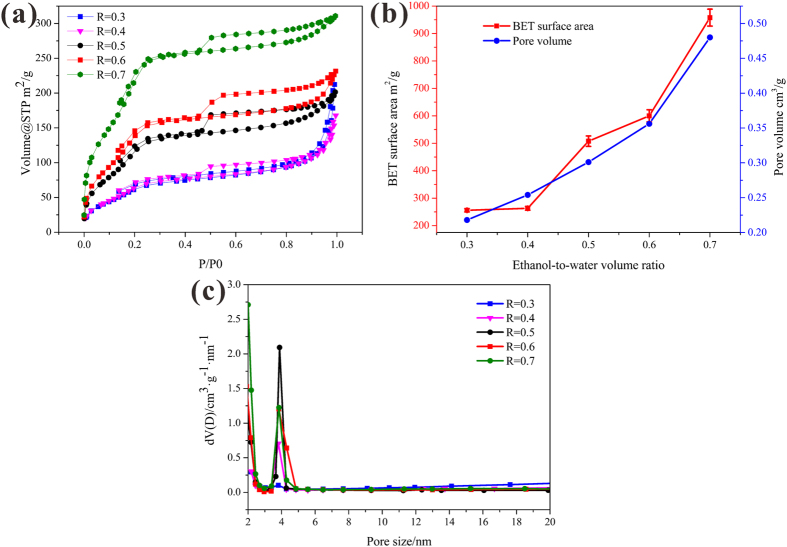
(**a**) Nitrogen sorption isotherms, (**b**) BET surface area and pore volume, (**c**) corresponding pore size distribution of HSS at different ethanol-to-water volume ratio (R_e/w_ = 0.3, 0.4, 0.5, 0.6 and 0.7, respectively).

The nitrogen sorption isotherms of HSS at different R_e/w_ in Fig. 8(a) exhibited typical IV curves with a N_2_ hysteresis loop that gradually closed at a partial pressure near 0.4, revealing characteristics of obtained HSS with high surface areas and narrow pore size distribution^45^. The BET surface area and pore volume of HSS were calculated and compared in Fig. 8(b), which obviously indicated a gradual increase in specific surface area and pore volume of HSS when the R_e/w_ changed from 0.3 to 0.7. For the optimization of HSS (R_e/w_ = 0.6), the BET surface area and the pore volume were found to be 600.05 m^2^/g and 0.356 cm^3^/g, respectively. When R_e/w_ was up to 0.7, the BET surface area and the pore volume were calculated to be as high as about 957.97 m^2^/g and 0.480 cm^3^/g, both of them are larger than R_e/w_ = 0.6. At the same time, the detailed pore size distribution calculated based on the BJH method suggested that HSS prepared at different R_e/w_ have uniform mesoporous size of about 3.8 nm and narrow pore size distribution ranging from 3.2 to 4.5 nm (Fig. 8c). Considering the aforementioned results that the diameter of HSS maintained a stable value but the hollow interior cavity decreased at R_e/w_ = 0.7. The highest surface area of HSS at R_e/w_ = 0.7 can probably be attributed to the greater numbers of pores in the shells.

### Growth mechanism of HSS

**Figure 9:**
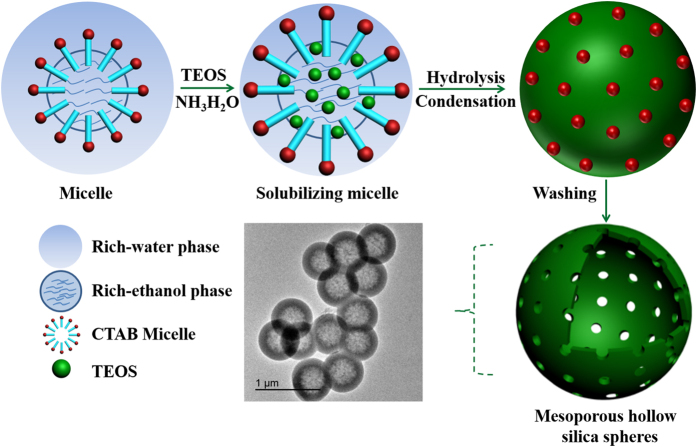
Schematic illustration of the formation processes of HSS.

Based on above morphological changes observed via the SEM images, an inward-growth mechanism involving sol-gel and electrostatic self-assembly of HSS was proposed in Fig. 9. First and foremost, cationic surfactant formed micelles in appropriate volume ratios of ethanol-water systems. Especially, a rich-ethanol phase was inside micelles and a rich-water phase was outside micelles owing to their differences in dielectric constant. Upon adding TEOS under stirring action, they would diffuse into the internal hydrophobic region of CTAB micelles together with ethanol because of their hydrophobic properties, they form an “oil-in-water” emulsion, in which the “oil” is TEOS in rich ethanol phase inside the micelles, and the “water” is rich-water phase outside the micelles. The “oil-in-water” emulsion act as a temporary template to define the morphology of HSS. Then ammonia catalyst was added to adjust the pH value and provide ideal reaction condition for silicon source. Lastly, under the catalysis of ammonia, the TEOS inside micelles hydrolyzed and condensed rapidly to form silica seeds^46^,^47^. During the sol-gel process, silica seeds with negative charge are drawn expeditiously to the hydrophilic end of cationic micelles by electrostatic interaction, which induced the synthesis of silica shell on the inner surface of the micelle. Since the hydrophobic interactions, the internal non-hydrolyzed TEOS will remain inside micelle interior until completion of hydrolysis. Although the silica shell formed earlier is loose with surface defects, the resulting silica seeds furtherly deposited inward, with continual increase in hollow silica shell thickness.

Inferring from above, we draw information that the amount of TEOS and reaction time would certainly determine the shell thickness but has had no effect on particle size. Remarkably, an appropriate amount of ethanol can stabilize the template, so that the silica condensation can take place without interruption. Ethanol can also enter into micelles to expand the template, producing larger silica spheres with hollow interior. However, it is worth noting that when an excess amount of ethanol was added, they would spread into water phase due to the excellent inter solubility of ethanol and water. This leads to the increased solubility of TEOS in rich-water phase outside the micelles, resulting in smaller HSS formation.

### Structure of WPU/HSS composite membrane

**Figure 10:**
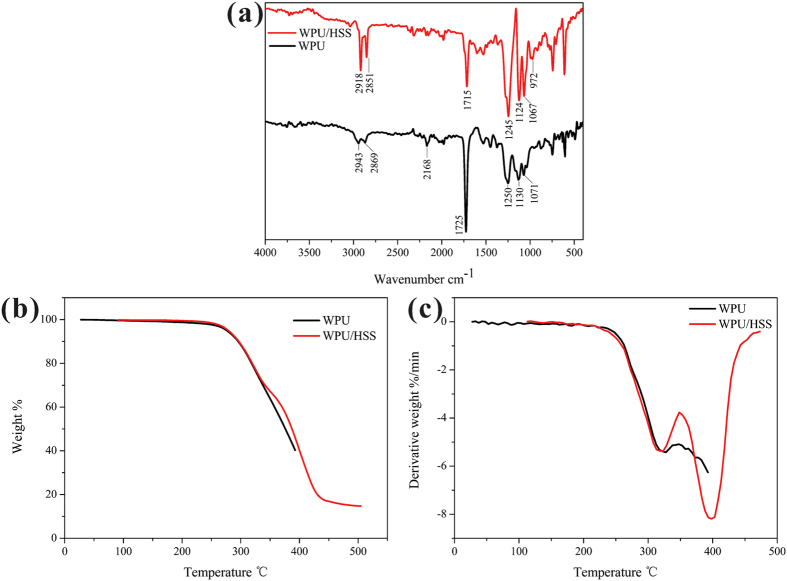
(**a**) FT-IR spectra, (**b**) TGA curves and (**c**) DTG curves of WPU membrane and WPU/HSS composite membrane.

In order to understand the interaction force between WPU chains and HSS, the FT-IR measurements of composite membrane were carried out. As shown in Fig. 10(a), the FT-IR spectrum of WPU membrane showed two apparent peaks at 1725 cm^−1^ and 1250 cm^−1^, which can be assigned to the stretching vibration of C = O group and C-N group respectively, clearly indicating the characteristic frameworks in a hard segment of PU. While the peak position of C = O group in the FT-IR spectrum of WPU/HSS was at 1715 cm^−1^, this shift suggested that there were some strong interactions in C = O and Si-OH of WPU/HSS composite membrane. Additionally, the band located at 972 cm^−1^ was associated with the Si-OH stretching vibration, which implied the existence of HSS in the composite membrane. However, compared to the bending vibration absorption of Si-OH located at 964 cm^−1^ in FT-IR spectrum of HSS (Fig. 3a), this slight shift was also attributed to the interaction between C = O and Si-OH. Figure 10(b,c) presented the typical TGA and DGT curves of WPU and WPU/HSS composite membrane. The weight loss in temperature range of 350–500 °C was shown that the thermal decomposition behavior of WPU/HSS composite membrane slightly shifted toward the higher temperature range compared with that of pure WPU. This confirmed the enhancement of thermal stability of hybrid WPU. The hybrid system may have an interpenetrated network nature and lead to the increase in the thermal stability, due to HSS acted as restriction sites to limit the segmental movement of WPU chains^48^. These above analyses indicate that rich hydroxyl groups on the surface of HSS could be interacted with WPU chains via H-bond force.

### The influence of ethanol-to-water volume ratio on the morphology of WPU/HSS composite membrane

**Figure 11:**
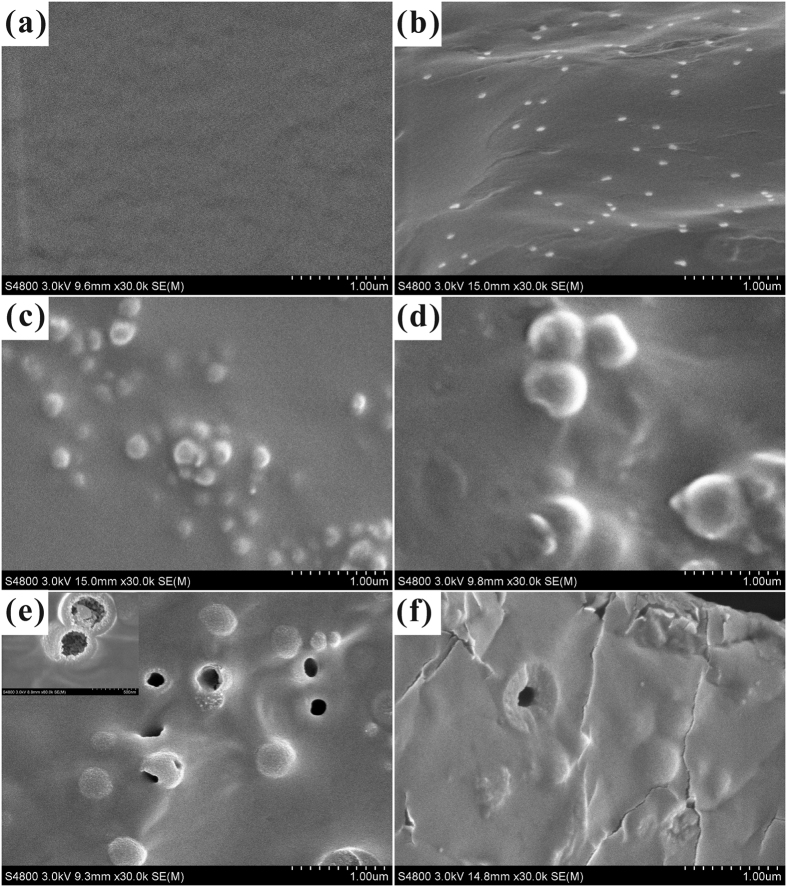
SEM images of (**a**) WPU membrane and (**b–f**) WPU/HSS membranes, in which HSS was prepared at different ethanol-to-water volume ratio: (**b**) 0.3, (**c**) 0.4, (**d**) 0.5, (**e**) 0.6, (**f**) 0.7.

**Figure 12:**
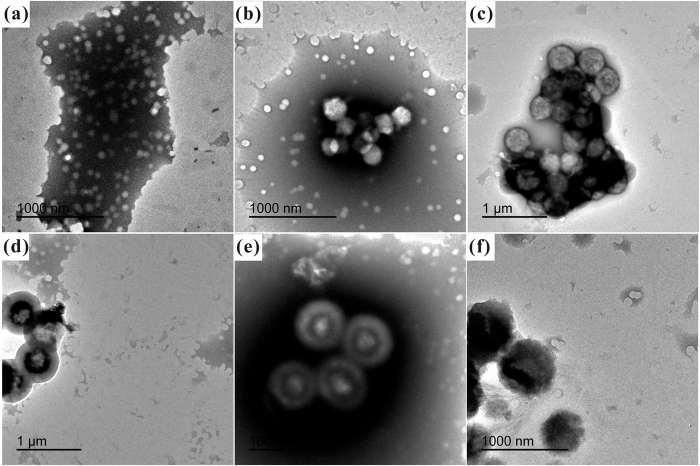
TEM images of (**a**) WPU membrane and (**b–f**) WPU/HSS membranes, in which HSS was prepared at different ethanol-to-water volume ratio: (**b**) 0.3, (**c**) 0.4, (**d**) 0.5, (**e**) 0.6, (**f**) 0.7.

In order to further test the dispersibility of HSS in polymer matrix, the morphology of the as-prepared hybrid materials was examined by SEM and TEM. As shown in Fig. 11, we could clearly identify that a certain number of HSS dispersed in the cross-section of WPU membrane, showing a variant individual dispersion at different R_e/w_. When R_e/w_ was 0.6 (Fig. 11e), as expected, the as-synthesized HSS possessed apparent internal hollow cavity and better monodispersion in WPU. Moreover, it also can be revealed from the TEM images as shown in Fig. 12, severe aggregation was observed when HSS made at R_e/w_ = 0.4 (Fig. 12c), which was consistent with the SEM study. With the increasing of R_e/w_, HSS indicated good individual dispersion in WPU and remained a clear hollow microstructure simultaneously. These facts demonstrate that the as-made WPU/HSS composite membrane would be suitable for the permeability of water vapor.

### Performance of WPU/HSS composite membrane

**Figure 13:**
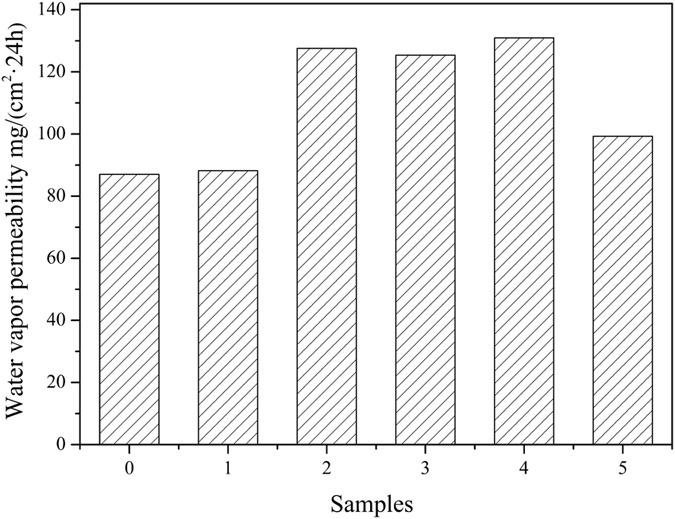
The effect of HSS prepared at different ethanol-to-water volume ratios on the water vapor permeability of WPU/HSS composite membrane. (0-WPU membrane, 1-5-WPU/HSS composite membrane, in which HSS prepared at R_e/w_ of 0.3, 0.4, 0.5, 0.6 and 0.7, respectively).

**Figure 14:**
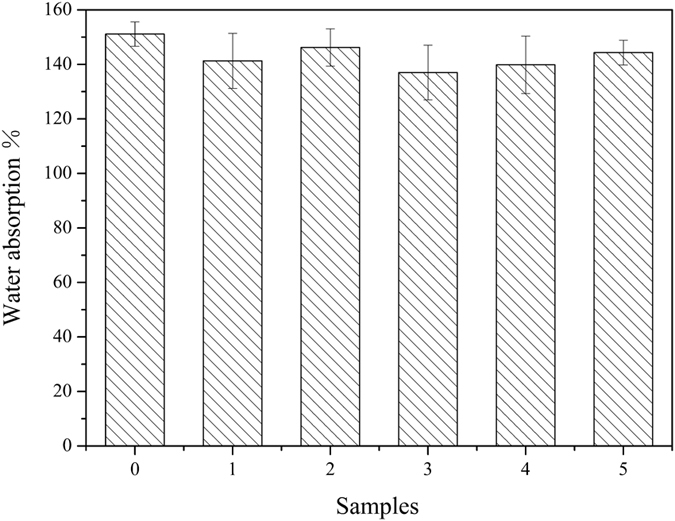
The effect of HSS prepared at different ethanol-to-water volume ratios on the water uptake of WPU/HSS composite membrane. (0-WPU membrane, 1-5-WPU/HSS composite membrane, in which HSS prepared at R_e/w_ of 0.3, 0.4, 0.5, 0.6 and 0.7, respectively).

**Figure 15:**
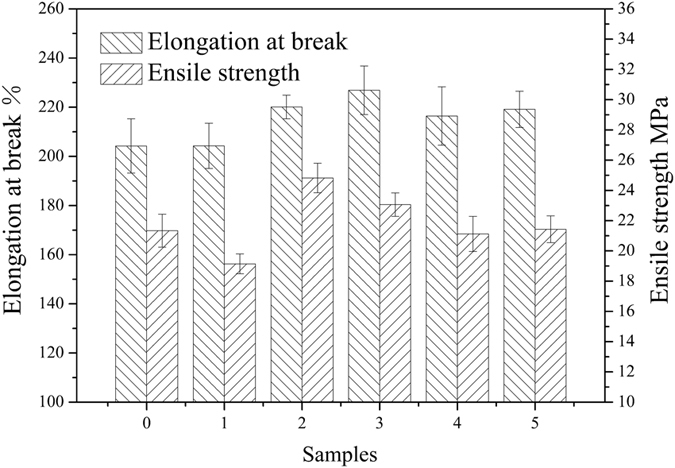
The effect of HSS prepared at different ethanol-to-water volume ratios on the mechanical properties of WPU/HSS composite membrane. (0-WPU membrane, 1-5-WPU/HSS composite membrane, in which HSS prepared at R_e/w_ of 0.3, 0.4, 0.5, 0.6 and 0.7, respectively).

HSS were introduced into WPU resin by physical blends to prepare WPU/HSS composite membrane. To make the HSS well-dispersed into polymer matrix for performance improvement of WPU, ultrasonic processing was carried out. Figures 13, 14, 15 showed the performances of composite membrane when 1 wt% HSS prepared at different R_e/w_ was incorporated into WPU. In comparison with pure WPU membrane, water vapor permeability, water resistance and mechanical properties of WPU/HSS composite membrane have been improved.

**Figure 16:**
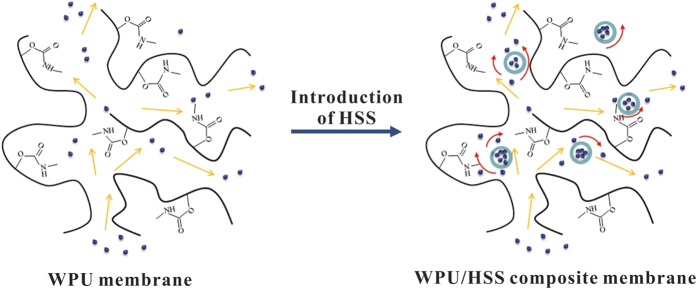
Schematic illustration of water vapor molecules penetrating through WPU/HSS composite membrane.

Traditional view that the size and shape of free-volume holes available in membrane materials control its permeability is generally accepted^49^. According to the measurement principle, the porosity and permeability of composite membrane are related directly to the water vapor permeability. Following the “adsorption-diffusion-desorption” mechanism, the diffusion of water vapor molecules through membrane is via both the organic-inorganic interface and the pore of HSS (Fig. 16). HSS with large specific surface area and hollow interior offered a relatively “free” pathway for water vapor molecules diffusion from the inner surface to the outside surface of composite membrane. The introduction of HSS into WPU can disrupt network of polymer chains and shorten the diffusion path length of water vapor molecules, making them diffuse quickly from the WPU-HSS organic-inorganic interface. Also, due to the amorphous structure of HSS, water vapor molecules can pass through the silica shell and be stored in the internal cavity, causing the vapour pressure differences between inside and outside surfaces of composite membrane. When the water vapor pressure is high enough, water vapor molecules spread rapidly out of the silica shell to the outer surface of membrane, improving the water vapor permeability of composite membrane remarkably. In our previous research, we have reported HSS with smaller hollow size are conducive to increase the water vapor permeability of composite. However, the water vapor permeability decreased sharply when the size of HSS is getting very small^6^. This can be illustrated by the reunion of small HSS, which decreased their dispersion degree in composite. Herein, when the R_e/w_ was 0.3, the obtained HSS with small particle diameter and unconspicuous hollow structure would agglomerate when blended with WPU, which hindered the transmission of water vapor molecules. By increasing the R_e/w_, the cavity volume of HSS became larger, which is more conducive to the diffusion of water vapor molecules, showing enhanced water vapor permeability (Fig. 13). In addition, the bigger the specific surface area of additives is, the better the water vapor permeability of membrane will be^49^. As the R_e/w_ increased to 0.7, the diameter of HSS remains nearly unchanged, but the cavity volume of HSS sharply reduced instead of further increased. Eventhough specific surface area is higher, the hollow interior cavity seem to have a more significant impact on the properities. So, the water vapor permeability of WPU/HSS composite membrane slightly decreased.

Water molecules hardly penetrate through HSS without the effect of water vapor pressure, diffusing mainly through the free volume between polymer chains. Therefore, the mesoporous shell of HSS can allow water vapor molecules to pass through while act a barrier to water molecules. The absorbed water can only exist in the space not occupied by hollow silica. In addition, the introduction of hollow silica spheres would limit the swing amplitude of polymer chain and thereby the free volume between polymer chains decreased. Hence, the diffusion of water molecules in membrane is hindered and the total water absorption is decreased (Fig. 14).

Compared to the pure WPU membrane, ensile strength and elongation at break of the composite membrane has been improved (Fig. 15). This could be ascribed to the reinforcing and toughening effect of nanoparticles on polymer matrix. Due to the large specific surface area and rich hydroxyl groups on their surface, the HSS could be interacted with WPU chains via physical bond and acted as restriction sites for the movement of polymer chains. Thus the composite membrane incorporated with HSS showed high resistance against the plastic deformation and reinforced mechanical performance. However, agglomeration of HSS would cause stress concentration and weakened reinforcement in polymeric matrix. So introducing the HSS prepared at R_e/w_ of 0.3 into WPU decreased the mechanical performance of composite membrane slightly.

## Conclusions

In summary, we successfully prepared HSS by micelle-template method using CTAB micelles as soft template and TEOS as silica precursor. The HSS possess a significant cavity, regular structure, narrow particle size distribution and amorphous shell with mesoporous structure. Particularly, the mechanism for formation of HSS was illustrated in detail and showed that the way of growth was from outside to inside along micelle template. Size of HSS increased gradually with the increase of R_e/w_ within a proper range due to the lower dielectric constant of ethanol, which can not only stabilize but also dissolve TEOS. Importantly, when HSS were introduced into WPU, rich hydroxyl groups on the surface of HSS could be interacted with WPU chains via H-bond force and showed good individual dispersion in WPU membrane. Performance tests indicated that the introduction of HSS can significantly improve the water vapor permeability of composite membrane, promoting its water resistance and mechanical performance at the same time. In future developments, the HSS/WPU hybrid membranes with excellent properties, in principle, could be as multifunctional coating and favorable for the actual production.

## Additional Information

**How to cite this article**: Bao, Y. *et al*. Micelle-template synthesis of hollow silica spheres for improving water vapor permeability of waterborne polyurethane membrane. *Sci. Rep.* **7**, 46638; doi: 10.1038/srep46638 (2017).

**Publisher's note:** Springer Nature remains neutral with regard to jurisdictional claims in published maps and institutional affiliations.
